# A Meta-Analysis of the Effects of Slow-Release Urea Supplementation on the Performance of Beef Cattle

**DOI:** 10.3390/ani10040657

**Published:** 2020-04-10

**Authors:** Saheed A. Salami, Colm A. Moran, Helen E. Warren, Jules Taylor-Pickard

**Affiliations:** 1 Solutions Deployment Team, Alltech (UK) Ltd., Stamford PE9 1TZ, UK; 2 Regulatory Affairs Department, Alltech SARL, Rue Charles Amand, 14500 Vire, France; cmoran@alltech.com; 3 Alltech Biotechnology Centre, Summerhill Road, A86 X006 Dunboyne, Ireland; hwarren@alltech.com (H.E.W.); jpickard@alltech.com (J.T.-P.)

**Keywords:** beef cattle, rumen degradable protein, urea, growth performance, feed efficiency

## Abstract

**Simple Summary:**

Plant-based feedstuffs, such as soybean meal and rapeseed meal are utilised to supply rumen degradable protein (RDP) and rumen undegradable protein (RUP) in the diets of ruminant animals (e.g., cattle, buffalo, sheep and goat). The RDP is utilised by rumen microbes for microbial protein synthesis. Microbial protein can contribute up to 80% of the protein requirement of ruminants. However, the use of plant protein sources in ruminant diets can be restricted based on their availability, costs and associated environmental impacts. Slow-release urea (SRU) is a non-protein nitrogen (NPN) source that allows for a partial replacement of vegetable RDP sources in ruminant diets by providing a sustained availability of ammonia for rumen microbial synthesis. Over the past three decades, extensive research has been conducted on the use of SRU in beef cattle diets. The current study analysed a combined dataset obtained from multiple research studies to derive quantitative and research-based evidence on the impact of a commercial SRU (Optigen^®^) on beef cattle performance. Results revealed that dietary supplementation of SRU improves the performance, profitability and environmental impacts of beef cattle production. Thus, this study demonstrates SRU as an effective NPN solution in beef cattle diets.

**Abstract:**

Slow-release urea (SRU) is a coated non-protein nitrogen (NPN) source for ruminant nutrition. This study applied a meta-analytic technique to quantify the effect of a commercial SRU (Optigen^®^, Alltech Inc., Nicholasville, KY, USA) on the performance of beef cattle. Data were extracted from 17 experiments and analysed using the random-effects model to estimate the effect size of SRU on dry matter intake (DMI), crude protein intake (CPI), live weight gain (LWG) and feed efficiency (FE) of growing and finishing beef cattle. There was no effect of feeding SRU on the overall DMI and CPI of beef cattle. Dietary inclusion of SRU improved the overall LWG (+92 g/d/head) and FE (+12 g LWG/kg DMI/head) of beef cattle. Notably, SRU supplementation in growing cattle exhibited a better improvement on LWG (130 vs. 60 g/d/head) and FE (18 vs. 8 g LWG/kg DMI/head) compared with finishing cattle. Moreover, SRU showed consistent improvements on the LWG and FE of beef cattle under several study factors. Simulation analysis indicated that positive effects of SRU on LWG and FE improved profitability through reduction in feed cost and reduced the emission intensity of beef production. These results indicate that SRU is a sustainable NPN solution in beef cattle production.

## 1. Introduction

The livestock industry is confronted with the challenges of using limited land and water resources to meet the growing demand for animal protein in an environmentally sustainable way. Ruminants are important components of a sustainable livestock sector because of their ability to digest and convert human inedible biomass to high-quality edible protein (meat and milk), primarily due to the intricate consortium of microbes residing in their rumen [1]. Crude protein (CP) in ruminant nutrition comprises of the rumen degradable protein (RDP) and rumen undegradable protein (RUP) fractions. Dietary RDP is degraded in the rumen to produce ammonia, which is synchronised with fermentable energy for rumen microbial growth and protein synthesis [2]. Microbial crude protein (MCP) and RUP reaching the small intestine constitute the metabolizable protein absorbed to meet the protein requirement of ruminants [3]. Microbial protein accounts for 50% to 80% of the total absorbable protein, highlighting its significance as a crucial component of metabolizable protein [2,4].

Dietary RDP is derived from nitrogenous compounds, which comprise of both non-protein nitrogen (NPN) sources and soluble true protein from plant and animal protein sources. The NPN sources are typically less expensive than true protein sources and feed-grade urea is often the most available NPN source used in ruminant diets. However, dietary utilisation of urea is limited due to its rapid hydrolysis to ammonia, exceeding the rate of carbohydrate fermentation in the rumen [2]. The lack of synchronisation between rumen ammonia production and fermentable energy availability negatively affect the efficiency of MCP yield. Consequently, this condition reduces the amount of MCP outflow, which may decrease the availability of metabolizable protein for production purposes in ruminants [5]. The rapid ruminal hydrolysis of urea may elevate blood ammonia concentration and increase the risk of ammonia toxicity and related negative health impacts in ruminants [6]. Furthermore, rapid ruminal hydrolysis of NPN sources, including urea, could reduce nitrogen (N) utilisation efficiency and thus increase N excretion and ammonia volatilisation from manure resulting in negative environmental impacts [5].

Over the last three decades, coating technology has been utilised to produce slow-release urea (SRU) products that degrade less rapidly in the rumen with potential claims of improved synchronisation of ruminal ammonia with energy digestion for microbial protein synthesis. These products are usually available for feeding to all ruminant species (cattle, buffalo, sheep and goat). Cherdthong and Wanapat [2] provided a narrative review of scientific literature that highlighted the potential efficacy of SRU in enhancing the efficiency of rumen N capture, microbial protein synthesis, fibre digestion and improved ruminant production. Moreover, SRU could be an eco-friendly alternative for replacing a portion of vegetable protein sources and the slow formation of ammonia in the rumen could ensure no negative impacts on N excretion [7,8]. However, narrative reviews lack methodological approach and they are subjective to the author’s interpretation of previous research, which may lead to biased conclusions [9].

It is often difficult to draw quantitative conclusions from the comparison of different research outcomes due to the diversity in study designs, experimental bias, poor statistical analysis and lack of application to a specific livestock production system [9]. These challenges can be overcome by the use of a meta-analysis, which is a rigorous statistical procedure for analysing a combined dataset obtained from multiple research studies [9,10]. A multitude of studies has utilised meta-analysis to provide quantitative and research-based evidence on the efficacy of nutritional products or interventions in beef cattle production [10,11,12]. To our knowledge, there is no published meta-analysis on the effect of SRU supplementation in beef cattle production. Thus, the objective of this study was to apply a meta-analytic technique to quantify the effects of SRU on the performance of growing and finishing beef cattle. In addition, the meta-analysis results were used to conduct a simulation analysis to evaluate the potential effect of SRU on the economic and environmental impacts of beef production. 

## 2. Materials and Methods 

### 2.1. Literature Search and Study Selection

Published and unpublished trial reports evaluating the effect of a commercial SRU (Optigen^®^, Alltech Inc., Nicholasville, KY, USA) on beef cattle were retrieved from the Alltech’s internal bibliography database. The SRU product comprises of urea evenly coated with a semi-permeable vegetable fat matrix containing 88% urea (41% N, 256% CP) and 11–12% fat. The fat coating in the SRU slows the dissolution of urea, resulting in a reduction in the rate of conversion of urea to ammonia in the rumen [13]. The unpublished trials are linked to the company’s research team, which allows for retrieving more information if required. An additional literature search was conducted on online academic databases and search engines (Google Scholar, Agricola and Pubmed) using the keywords: “coated-urea, Optigen^®^, beef, cattle, steer and performance”. There was no date restriction placed on the search engines, to encompass the entire duration Optigen^®^ has been utilised as an SRU supplement in beef cattle research. A total of 48 studies were obtained from the initial literature search and they were subjected to selection screening according to the following inclusion criteria: (1) the experiment was reported in English language and conducted in beef cattle or dairy beef cattle breeds; (2) information on the beef cattle production phase was provided or able to be discerned; (3) studies contain at least one control diet without SRU supplement and a diet supplemented with Optigen^®^ as the SRU; (4) details of feed composition and dosage of SRU supplemented were provided; (5) information on experimental period was reported; (6) information on experimental unit in study design (pen or individual feeding) was provided; (7) one or more performance parameters—dry matter intake (DMI), live weight gain (LWG) and feed efficiency (FE)—are reported. The screening process resulted in the selection of 17 experimental studies. The selected studies consist of seven peer-reviewed publications and 11 unpublished studies presented in a PhD dissertation or international conferences. The description of experimental studies included in the meta-analysis database is reported in Appendix A (Table A1). Table S1 presents the reference list of trials in which the SRU was fed to beef cattle but excluded from the meta-analysis due to failure to meet the inclusion criteria.

### 2.2. Data Extraction

A spreadsheet was developed to extract data from the studies. The following performance outcomes were extracted or calculated from each study for estimation of effect size: DMI, dietary protein intake (DPI), LWG and FE. Dietary protein intake was calculated by multiplying DMI and the level of protein in the diet for each treatment. Feed efficiency was reported as LWG/DMI in most of the experiments while few experiments reported FE as DMI/LWG. For these few experiments, FE was recalculated by dividing LWG with DMI for each treatment. The standard deviation (SD) was recorded as the measure of variance. If SD was not reported in studies, it was calculated by multiplying the reported standard error (SE) of means by the square root of the sample size. Many studies reported a common SE or SD and these estimates were used for both control and treatment groups. However, few trial reports provided a separate estimate of SD or SE for each group and these were recorded as such. The SD from other studies was imputed for a few performance outcomes that did not report the measure of variance. This approach has been supported by empirical evidence indicating the validity of substituting few missing variance data with reported variance data from another meta-analysis or other studies in the same meta-analysis to provide accurate meta-analysis results [14,15]. Information on the diversity of study factors that could influence the performance outcomes were included in the database. These include the production phase (growing or finishing cattle), peer-review (refereed journal or not), study location (North America or elsewhere), breed (European beef cattle or not), grouping method for feeding (individual or pen feeding), inclusion of corn silage as roughage source (yes or no), feeding period (≤80 or >80 d), SRU dosage (≤1.00% or >1.00% dry matter (DM) diet), Sex (steers, heifers, bull). 

### 2.3. Calculations and Statistical Analysis

Statistical analysis was performed using the Comprehensive Meta-analysis software (version 3, Biostat Inc., USA). A random-effects model was adopted for the meta-analysis, which has an underlying assumption that the distribution of effects exists, resulting in heterogeneity among study results [16]. The effect size for the analysed outcomes was determined as raw mean difference (RMD) and standardised mean difference (SMD) at a 95% level of confidence intervals. The RMD was estimated as the sum of the mean differences of treatment relative to control in the individual studies, weighted by the individual variances for each study. The RMD estimates the actual effect of treatment in unit measures. On the other hand, the SMD is the mean difference between treatment and control groups, which is standardised based on the SD of treatment and control groups and the result is a numerical dimensionless value. The SMD offers the advantage of being a more robust effect size estimate when there is heterogeneity in the dataset [17]. Significance of effect size estimates (RMD and SMD) was declared at *p* ≤ 0.05. Forest plots were used to visually present the effect size outcomes (RMD at a 95% confidence interval) from each study and the overall outcome of all the studies. 

Variations across studies were assessed using the chi-squared (*Q*) test and *I^2^* [16]. Considering that the *Q* test is presumed to have a relatively low power of detecting heterogeneity in meta-analytical studies, *I^2^* statistic is computed to describe the percentage of total variation across studies, which is due to heterogeneity rather than chance. The following equation was used to calculate the *I^2^* heterogeneity statistic from *Q*, where *k* is the number of trials:

(1) $$I^{2\;}=\frac{Q\;-\;(k\;-\;1)}{Q}\;\times \;100$$

If *I^2^* exceeds 50%, the outcome is presumed to have significant heterogeneity [17]. To control for heterogeneity, the studies were stratified into groups/subgroups based on different study factors (e.g., production phase, study location, breed, feeding period etc.) that could influence the performance outcomes and respective meta-analysis was performed to determine the effect size estimates. Notably, sub-groups with less than 10 comparisons are excluded from meta-analysis due to the general criticism that such model outcomes can be statistically biased [16].

Although a meta-analysis will yield a mathematically accurate synthesis of the studies included in the analysis, if these studies are a biased sample of all relevant studies, then the mean effect computed by the meta-analysis will reflect this bias. This issue is generally known as publication bias. In this study, publication bias was examined both graphically with funnel plots and statistically, using both the Begg’s test [18] and Egger’s test [19]. For example, if there is a bias because smaller studies without statistically significant effects remain unpublished, this will lead to an asymmetrical appearance of the funnel plot and a gap will be evident in a bottom corner of the graph. In this situation, the effect calculated in a meta-analysis will tend to overestimate the intervention effect [10].

### 2.4. Simulation Analysis

A simulation analysis was performed using the meta-analysis results to evaluate how the effects of SRU on beef cattle performance would influence the economic and environmental impacts of beef cattle production. The simulation was based on the impacts of feeding SRU to raise 1000 growing-finishing beef cattle to gain a 200 kg live weight (LW). Data used for the simulation inputs are presented in Appendix A (Table A2). The variables in the simulation input include the number of cattle, DMI, LWG, FE, target live weight gain, lean meat yield and beef protein output. Days on feed to slaughter and total feed cost (€/1000 cattle) were calculated as indicators for the economic impacts SRU on beef production. Emission intensity attributed to feed use (EIAFU) and total emission intensity were calculated as indicators of the environmental footprint of beef production. The following equations were applied to the simulation inputs to calculate the economic and environmental outputs for the baseline and SRU diets:

Ration required to gain 200 kg LW = Target live weight gain/Feed efficiency(2)

Days on feed to slaughter = Target live weight gain/Live weight gain(3)

Total feed use = Ration required to gain 200 kg LW × (1/DM content of diet)(4)

Feed cost = Total feed use × ration cost(5)

Total feed cost (€/1000 cattle) = Feed cost × 1000(6)

EIAFU = Beef protein output × AEIAFU(7)

Emission intensity = EIAFU + (AEINAFU × Beef protein output)(8)

Total emission intensity = Emission intensity × 1000(9)

where EIAFU is the emission intensity attributed to feed use, AEIAFU is the global average emission intensity attributed to feed use and AEINAFU is the global average emission intensity not attributed to feed use.

The following assumptions were applied to the equations above:

1. DM content of diet = 70%
2. Ration cost = 0.15 €/kg as-fed. Ration cost was assumed to be similar for the baseline and SRU diets considering that diets can be reformulated with SRU by replacing a portion of the vegetable protein sources, such as soybean meal without changing the feed cost under several circumstances. Thus, no additional cost is attributed to the SRU diet in this scenario.
3. AEIAFU = 108 kg CO_2_-eq per kg of protein. This assumption was based on reported data, which indicated that the global average emission intensity of beef is 300 kg CO_2_-eq per kg of protein and an average of 36% of beef emissions was attributed to feed use [20]. Data on the global average emission intensity of beef was used because of the vast diversity in the environmental footprint of beef production systems across the world.
4. AEINAFU = 192 kg CO_2_-eq per kg of protein. Based on Assumption (3) above, the remaining component of beef emissions was allocated to non-feed use.

## 3. Results

### 3.1. Study Characteristics

A summary of the studies used for this meta-analysis is provided in Table A1. The studies were conducted in nine countries (six from the US; two from Uruguay, Brazil and Mexico; one each from Italy, Egypt, Ireland, Portugal and Argentina) over a 17-year period (2002–2018). There were 32 control and SRU dietary comparisons for DMI, DPI and FE and 33 dietary comparisons for LWG. A vast majority (67%) of the trial comparisons were conducted in North America (USA and Mexico). The average SRU dose supplemented across all studies was calculated from the meta-analysis database as 0.88% DM diet. European beef cattle breeds and steers were used in 76% and 85% of all trial comparisons, respectively, and well-distributed across all study locations. Moreover, there was almost an equal proportion of studies conducted in growing cattle (45%) and finishing cattle (55%). Thus, this dataset gives a good representation to draw important conclusions from this meta-analysis while recognising the wide diversity in beef cattle production systems across different regions.

### 3.2. Effect of Slow-Release Urea on Dry Matter Intake of Beef Cattle

**Figure A1:**
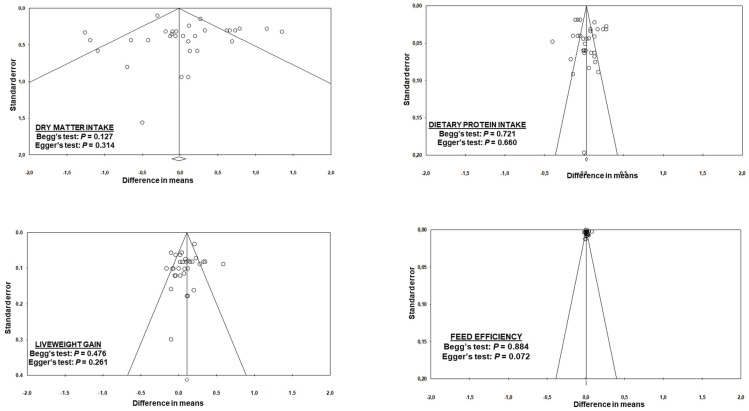
Funnel plots of raw mean differences (difference in means) against their inverse standard errors and the associated significance (*p*-value) for testing the publication bias of the studies included in the meta-analysis. Open circles represent individual study comparisons included in the meta-analysis.

There was no effect of SRU supplementation on the overall DMI of beef cattle as indicated by both effect size estimates, RMD and SMD (Table 1 and Figure S1). There was substantial variation across all studies as revealed with the *Q* and *I^2^* statistics in Table 1. However, none of the factors employed to stratify the studies showed a significant effect of SRU on the DMI of beef cattle (Table 1). The funnel plot, as well as the lack of significance of Begg’s and Egger’s tests (Appendix A, Figure A1), indicated that there was no publication bias, suggesting that enough studies were obtained to analyse the effect of SRU on the DMI of beef cattle. 

### 3.3. Effect of Slow-Release Urea on Dietary Protein Intake of Beef Cattle

The impacts of SRU supplementation on the DPI of beef cattle are presented in Table 2 and Figure S2. No significant effect of SRU on the overall DPI of beef cattle was found. However, there was variation in the DPI dataset across all studies. Stratification based on different study factors indicated that SRU significantly increased the DPI in studies conducted in growing beef cattle (+80 g/d/head), studies reported in peer-review journals (+82 g/d/head) and studies conducted in North America (+71 g/d/head). There was no indication of publication bias in the studies used for analysing the effect of SRU on the DPI of beef cattle (Appendix A, Figure A1). 

### 3.4. Effect of Slow-Release Urea on Live Weight Gain of Beef Cattle

The supplementation of SRU significantly increased the LWG of beef cattle by +92 g/d/head across all studies (Table 3 and Figure S3). The *Q* and *I^2^* statistics indicated that there was variation in the LWG dataset across all studies. Meta-analysis of stratified sub-groups showed that cattle fed SRU diets exhibited higher LWG in studies conducted in growing cattle (+130 g/d/head) and finishing cattle (+60 g/d/head), non-peer-review (+73 g/d/head) and peer-review studies (+115 g/d/head), studies conducted in North America (+100 g/d/head), studies using European beef cattle breeds (+119 g/d/head) and steers (+91 g/d/head), studies with individual animal feeding (+100 g/d/head), studies where corn silage was fed (+142 g/d/head), studies with ≤80 d feeding period (+101 g/d/head) and studies feeding ≤1% DM of SRU in the diet (+81 g/d/head). There was no publication bias in the studies used for analysing the effect of SRU on the LWG of beef cattle (Appendix A, Figure A1).

### 3.5. Effect of Slow-Release Urea on Feed Efficiency of Beef Cattle

Feeding SRU significantly enhanced the FE of beef cattle by +12 g LWG/kg DMI/head across all studies (Table 4 and Figure S4). There was a variation in the FE dataset across all studies as shown with the *Q* and *I^2^* statistics. Sub-group meta-analyses indicated that dietary inclusion of SRU increased the FE in studies conducted in growing cattle (+18 g LWG/kg DMI/head) and finishing cattle (+8 g LWG/kg DMI/head), non-peer-review (+7 g LWG/kg DMI/head) and peer-review studies (+16 g LWG/kg DMI/head), studies conducted in North America (+14 g LWG/kg DMI/head), studies using European beef cattle breeds (+15 g LWG/kg DMI/head) and steers (+13 g LWG/kg DMI/head), studies with individual animal feeding (+12 g LWG/kg DMI/head) and pen-level feeding (+12 g LWG/kg DMI/head), studies where corn silage was fed (+17 g LWG/kg DMI/head), studies with ≤80 (+11 g LWG/kg DMI/head) or >80 days feeding period (+14 g LWG/kg DMI/head) and studies feeding ≤1% DM of SRU in the diet (+11 g LWG/kg DMI/head). There was no publication bias in the studies used for analysing the effect of SRU on the FE of beef cattle (Appendix A, Figure A1).

### 3.6. Environmental and Economic Impacts of Feeding Slow-Release Urea in Beef Cattle Production

The simulation analysis indicated that the efficacy of SRU in improving LWG and FE of beef cattle improved the economic and environmental impacts of beef production. As shown in Table 5, feeding SRU to 1000 growing-finishing cattle to gain 200 kg live weight reduced the days on feed to slaughter by 9 d and resulted in a significant financial gain of €16,500 attributed to a reduction in feed cost. In addition, feeding SRU reduced feed emissions for beef production by 111.5 tons CO_2_-eq, contributing approximately 2.2% reduction in the carbon footprint of beef production.

## 4. Discussion

There is a crucial need for well-designed research studies with efficacy results that could support animal nutritionists and producers in making the most profitable decision on the choice of feeding technologies to apply in livestock operations. To our knowledge, the present study is the first to apply a meta-analytic technique to provide an objective review of the retrospective effect of SRU supplementation on the performance of beef cattle. The SRU evaluated in this study has an N content of 41%, which can supply an equivalent CP of 256% from an NPN source. This allows for reformulating diets with SRU in such a way that a lower inclusion level of vegetable protein sources, such as SBM (CP, 40–48%), can be achieved. Thus, the SRU concentrates the nitrogen fraction of the diet and supplies slowly degraded N to the rumen.

This provides sustained availability of ammonia in the rumen environment to synchronise fermentable energy for optimal microbial protein synthesis [21]. An improvement in rumen MCP is expected to increase the metabolizable protein supply and, therefore, enhance the production performance of ruminants [22]. Galyean [23] noted that supplemental protein from RDP sources compared to RUP sources produced more consistent results to improve the performance response of finishing beef cattle. Moreover, the authors emphasised the crucial need of accounting for the rumen microbial N need when estimating the protein requirements of beef cattle with a metabolizable protein system. Therefore, the application of SRU as an NPN source has attracted increased interest in ruminant nutrition to meets the rumen microbial N need. 

The results of this meta-analysis showed that supplementation of SRU did not affect the overall DMI and DPI of beef cattle across all trials. Several studies investigating the replacement of vegetable protein sources with SRU have demonstrated that SRU supplementation did not influence the DMI of beef cattle [24,25,26,27]. However, studies comparing uncoated urea with SRU in forage-based and TMR diets have shown that SRU diets increased the DMI of sheep and cattle due to increases in fibre digestion and nutrient digestibility [28,29,30]. Feed digestibility is considered as one of the significant physiological drivers of DMI as a consequence of the effect of feed digestibility in increasing the ruminal passage rate of digesta [31]. The lack of effect of SRU on feed digestibility could partly explain the non-significant effect on DMI when vegetable protein sources were partially substituted with SRU [25,26]. The level of DPI is determined by the DMI and dietary protein content, and any difference in either the former or latter or both would effect a proportional change in DPI. Thus, the lack of effect of SRU on overall DPI is partly a consequence of the non-significant effect on DMI. However, a higher DPI was observed in growing cattle fed SRU diets, which could be attributed to an increased protein level in the SRU diets (+0.9% DM diet; data not shown). 

Supplemental SRU increased the LWG and FE of beef cattle. However, LWG and FE considered across all studies was heterogeneous, suggesting that other dietary and animal management factors could influence the observed effect of SRU on LWG and FE. Nonetheless, the improvement in LWG and FE was consistently observed under several study factors as shown with the results of the group/sub-group stratification analyses. The positive effect of SRU on LWG and FE is possibly a response to improved synchronisation of ammonia and fermentable energy, which increased microbial protein synthesis and the supply of metabolizable protein for growth performance. Interestingly, the supplementation of SRU had a greater effect on LWG and FE when fed to growing beef cattle compared with finishing beef cattle. This could in part be attributed to the higher DPI in growing cattle, considering that protein is a crucial nutrient for growth. In addition, this may be attributed to possible differences in ration specifications fed between the two production phases. Energy-dense diets are usually fed in the finishing phase to accelerate maximum weight gain and meat yield whereas growing beef cattle are fed less-energy dense diets (generally based on fresh or conserved forages) to ensure a steady continuous frame growth. Thus, the impact of the finishing diets in maximising the growth rate of beef cattle could have exerted a subtle masking effect on the efficacy of SRU supplementation in the finishing cattle. Moreover, growing cattle diets based on fresh or conserved forages are prone to fluctuations in protein quality [32] and the efficacy of supplemental degradable N has been shown to be more pronounced in diets deficient in degradable intake protein and when metabolizable protein is limiting [2,33]. Additionally, cattle fed growing diets consume vegetable protein sources and roughages including the main energy source (corn silage), which have slower ruminal fermentation rates than the concentrate feeds used in the finishing phase. Thus, it is possible that synchronisation of carbohydrate fermentation and RDP supply from SRU was favoured in the growing cattle diets compared with the finishing diets. Furthermore, studies where corn silage was included as a forage source in the diet showed an improvement in LWG and FE compared to other forage sources. This suggests that the provision of corn silage as the roughage source is a useful dietary strategy to enhance the effect of SRU on beef cattle performance. Indeed, corn silage has been widely used as a forage source for ruminants because of its high digestible energy and digestibility value [34]. Thus, corn silage could have provided fermentable carbohydrate with better synchronisation with ammonia to optimise microbial protein synthesis when SRU is supplemented. In contrast, forage sources with low digestible energy content or slow fermentation rate may reduce the utilisation of ammonia for microbial protein synthesis. In an extensive review of *in vivo* studies, Givens and Rulquin [35] summarised that forage sources could influence nitrogen utilisation for rumen microbial protein synthesis. The authors noted that corn silage-based diets enhanced microbial protein synthesis compared to diets based on grass and legume silages. 

Several studies have evaluated graded levels of SRU supplementation with inconsistent results on the performance of beef cattle [26,27,36]. The variation in the utilisation of urea levels may be due to several factors, such as the continuous availability of fermentable carbohydrate, level of RDP and nitrogenous compounds in the basal diet, feeding regiment—*ad libitum* or restricted feeding—and adaptation to urea feeding [37]. In the present study, we stratified the studies into two groups (≤1.00% and >1% DM diet) based on the level of SRU included in the diet. Interestingly, studies that fed SRU at ≤1.00% DM diet consistently showed a significant increase in LWG and FE whereas no significant improvement was observed in beef cattle fed SRU at >1% DM diet. The average SRU dosage across all studies evaluated in this meta-analysis was 0.88% DM diet. In agreement with our observation, Shain et al. [38] demonstrated that supplementing urea above 0.88% DM (compared to 1.34% or 1.96% DM diet) in dry-rolled corn finishing diets had no beneficial effect on the performance of steers. For the commercial SRU evaluated in this study, the recommended dose to provide NPN in beef cattle diets is 10 g/kg DMI/d/head. In finishing beef cattle consuming 9.5 kg DMI, this recommended SRU dosage can be delivered at an inclusion level of <1.00% DM diet. This suggests that it would be possible to reformulate diets within the SRU dosage range that (≤1.00%) optimise cattle performance even in finishing beef cattle. 

Beef production has the largest environmental footprint compared with other livestock products, contributing approximately 41% of the greenhouse gas emissions of the global livestock sector [20]. There is a vast diversity in the efficiency of beef production systems, which creates opportunities for reducing the global environmental impacts of beef production [39]. Feed efficiency is increasingly recognised as a significant tool for reducing the environmental footprint of beef production due to its beneficial effect in improving resource use efficiency per unit product and reducing enteric methane emission [40,41]. Interestingly, there is a positive relationship between FE and environmental footprint and economic sustainability, suggesting that strategies that improve the FE of beef cattle could lead to a simultaneous improvement in environmental impacts and profitability of beef production [41]. Dietary strategies and nutritional technologies have been demonstrated as potential tools to improve FE and reduce the environmental impacts of livestock production [42,43,44]. In agreement with these assertions, the simulation analysis employed in this study showed that the positive effect of SRU on LWG and FE reduced the days on feed to slaughter and improved the profitability of beef production through a 6% reduction in feed cost. Similarly, the impact of SRU on LWG and FE reduced the environmental footprint of beef production by 2.2% (−111.5 tonnes CO_2_-eq) due to a reduction in emissions associated with feed use. In perspective, this carbon emission saving is equivalent to the carbon footprint of one person taking 134 transatlantic flights from London to New York or an annual average of 73 new cars taken off the road in the UK. It is noteworthy that further improvement in profitability and environmental impacts can be captured from the reduction in days on feed to slaughter. This is because decrease in days on feed to slaughter could result in lessening other economic and environmental costs, such as labour, fuel and energy inputs routinely associated with beef production. However, the additional benefits of reduced days on feed to slaughter were not evaluated in the current simulation. Thus, further studies based on whole-farm modelling would be required to investigate the holistic effect of SRU feeding on beef production at the farm-level.

## 5. Conclusions

These meta-analysis results showed that SRU supplementation exhibited a consistent improvement in the LWG and FE of beef cattle under several study factors. The inclusion of corn silage as the roughage source in cattle diets enhanced the positive effects of SRU on LWG and FE. The positive effects of SRU on beef cattle performance could improve the economic and environmental sustainability of beef production.

## Figures and Tables

**Table 1:** Summary of effect size estimates for dry matter intake (kg/d/head) of beef cattle fed control and SRU diets in a random-effect meta-analysis.

Group/Sub-Group ^1^	Number of Comparisons	Effect Size Estimates						Heterogeneity Tests		
		RMD (95% CI)	SE	*p*-Value	SMD (95% CI) ^3^	SE	*p*-Value	*Q*	*p*-Value	*I^2^* (%)
All trials	32	0.074 (−0.132, 0.281)	0.105	0.480	0.045 (−0.209, 0.299)	0.129	0.728	87.70	<0.001	64.65
Production phase										
Growing	15	0.238 (−0.043, 0.520)	0.144	0.097	0.269 (−0.171, 0.709)	0.225	0.231	43.82	<0.001	68.05
Finishing	17	−0.114 (−0.446, 0.217)	0.169	0.499	−0.111 (−0.432, 0.210)	0.164	0.498	43.49	<0.001	63.21
Peer-review										
No	15	0.032 (−0.266, 0.330)	0.152	0.833	0.030 (−0.264, 0.324)	0.150	0.839	36.86	0.001	62.01
Yes	17	0.111 (−0.186, 0.409)	0.152	0.464	0.076 (−0.399, 0.551)	0.242	0.753	50.66	<0.001	68.42
Study location										
North America ^2^	22	0.119 (−0.136, 0.374)	0.130	0.362	0.135 (−0.178, 0.449)	0.160	0.398	41.68	0.005	49.62
Breed										
European beef cattle ^3^	25	0.134 (−0.126, 0.395)	0.133	0.312	0.169 (−0.127, 0.466)	0.151	0.264	71.80	<0.001	66.58
Grouping method for feeding										
Individual	20	0.100 (−0.178, 0.379)	0.142	0.480	0.033 (−0.265, 0.332)	0.152	0.826	65.733	<0.001	71.10
Pen	12	0.041 (−0.261, 0.343)	0.154	0.791	0.077 (−0.441, 0.596)	0.264	0.770	21.938	0.025	49.86
Corn silage in diet										
No	14	−0.157 (−0.427, 0.113)	0.138	0.255	−0.110 (−0.357, 0.137)	0.126	0.382	14.95	0.310	13.05
Yes	18	0.217 (−0.058, 0.492)	0.140	0.123	0.233 (−0.168, 0.635)	0.205	0.255	68.74	<0.001	75.27
Feeding period (days)										
≤80	21	0.120 (−0.122, 0.361)	0.123	0.331	0.116 (−0.145, 0.377)	0.133	0.384	42.22	0.003	74.90
>80	11	0.011 (−0.338, 0.360)	0.178	0.950	−0.070 (−0.658, 0.518)	0.300	0.816	39.84	<0.001	52.63
SRU dosage (% DM diet)										
≤1.00	22	−0.015 (−0.257, 0.228)	0.124	0.906	−0.074 (−0.400, 0.252)	0.166	0.656	68.84	<0.001	69.49
>1.00	10	0.298 (−0.034, 0.629)	0.169	0.079	0.260 (−0.149, 0.669)	0.209	0.213	18.59	0.029	51.58
Sex										
Steers	27	0.031 (−0.193, 0.255)	0.114	0.786	−0.008 (−0.307, 0.291)	0.152	0.958	73.41	<0.001	64.58

^1^—Studies were stratified into group and subgroups by study factors that could influence performance outcome. Subgroups with <10 comparisons are excluded from analysis; ^2^—Studies were conducted in USA and Mexico; ^3^—These include: Charolais, Limousine, Hereford, Angus and Angus crossbred. SRU: slow-release urea; RMD: raw mean difference and its associated 95% confidence interval; SMD: standardised mean difference and its associated 95% confidence interval; SE: standard error. *Q*: chi-squared statistic and associated significance level (*p*-value); *I^2^*: percentage of variation.

**Table 2:** Summary of effect size estimates for dietary protein intake (kg/d/head) of beef cattle fed control and SRU diets in a random-effect meta-analysis.

Group/Sub-Group ^1^	Number of Comparisons	Effect Size Estimates						Heterogeneity Tests		
		RMD (95% CI)	SE	*p*-Value	SMD (95% CI)	SE	*p*-Value	Q	*p*-Value	I^2^ (%)
All trials	32	0.033 (−0.020, 0.085)	0.027	0.222	0.307 (−0.094, 0.708)	0.205	0.133	207.09	<0.001	85.03
Production phase										
Growing	15	0.080 (0.001, 0.159)	0.040	0.046	0.987 (0.201, 1.772)	0.401	0.014	113.31	<0.001	87.64
Finishing	17	−0.015 (−0.076, 0.046)	0.031	0.634	−0.110 (−0.544, 0.325)	0.222	0.621	80.39	<0.001	80.10
Peer-review										
No	15	−0.022 (−0.084, 0.040)	0.032	0.494	−0.158 (−0.610, 0.294)	0.231	0.493	86.93	<0.001	83.90
Yes	17	0.082 (0.005, 0.160)	0.040	0.038	0.978 (0.206, 1.750)	0.394	0.013	109.56	<0.001	85.40
Study location										
North America ^2^	22	0.071 (0.013, 0.129)	0.030	0.016	0.778 (0.231, 1.325)	0.279	0.005	109.31	<0.001	80.79
Breed										
European beef cattle ^3^	25	0.051 (−0.010, 0.112)	0.031	0.101	0.590 (0.109, 1.071)	0.245	0.016	178.17	<0.001	86.53
Grouping method for feeding										
Individual	20	0.013 (−0.054, 0.080)	0.034	0.697	0.099 (−0.346, 0.544)	0.227	0.663	140.00	<0.001	86.43
Pen	12	0.063 (−0.018, 0.144)	0.041	0.126	0.904 (−0.091, 1.900)	0.508	0.075	62.67	<0.001	82.45
Corn silage in diet										
No	14	0.035 (−0.005, 0.075)	0.021	0.088	0.239 (−0.078, 0.556)	0.162	0.140	23.24	0.039	44.07
Yes	18	0.035 (−0.041, 0.110)	0.039	0.368	0.448 (−0.240, 1.136)	0.351	0.202	177.14	<0.001	90.40
Feeding period (days)										
≤80	21	0.049 (−0.008, 0.107)	0.030	0.094	0.396 (−0.018, 0.809)	0.211	0.061	100.63	<0.001	90.06
>80	11	0.004 (−0.098, 0.106)	0.052	0.938	0.087 (−0.927, 1.100)	0.517	0.867	102.20	<0.001	80.43
SRU dosage (% DM diet)										
≤1.00	22	0.014 (−0.038, 0.067)	0.027	0.597	0.138 (−0.332, 0.608)	0.240	0.564	139.59	<0.001	86.27
>1.00	10	0.071 (−0.035, 0.177)	0.054	0.187	0.826 (−0.029, 1.680)	0.436	0.058	65.57	<0.001	84.96
Sex										
Steers	27	0.030 (−0.030, 0.090)	0.031	0.325	0.335 (−0.166, 0.837)	0.256	0.190	189.85	<0.001	86.31

^1^—Studies were stratified into group and subgroups by study factors that could influence performance outcome. Subgroups with <10 comparisons are excluded from analysis. ^2^—Studies were conducted in USA and Mexico. ^3^—These include: Charolais, Limousine, Hereford, Angus and Angus crossbred. SRU: slow-release urea; RMD: raw mean difference and its associated 95% confidence interval; SMD: standardised mean difference and its associated 95% confidence interval; SE: standard error. *Q*: chi-squared statistic and associated significance level (*p*-value); *I^2^*: percentage of variation.

**Table 3:** Summary of effect size estimates for live weight gain (kg/d/head) of beef cattle fed control and SRU diets in a random-effect meta-analysis.

Group/Sub-Group ^1^	Number of Comparisons	Effect Size Estimates						Heterogeneity Tests		
		RMD (95% CI)	SE	*p*-Value	SMD (95% CI)	SE	*p*-Value	*Q*	*p*-Value	*I^2^* (%)
All trials	33	0.092 (0.037, 0.147)	0.028	0.001	0.354 (0.126, 0.581)	0.116	0.002	72.51	<0.001	55.87
Production phase										
Growing	15	0.134 (0.036, 0.232)	0.050	0.007	0.653 (0.183, 1.123)	0.240	0.006	48.23	<0.001	70.97
Finishing	18	0.060 (−0.002, 0.121)	0.031	0.056	0.310 (0.088, 0.532)	0.113	0.006	23.49	0.134	27.64
Peer-review										
No	16	0.073 (0.016, 0.129)	0.001	0.011	0.386 (0.193, 0.579)	0.098	<0.001	18.56	0.234	19.18
Yes	17	0.115 (0.014, 0.217)	0.003	0.026	0.564 (0.076, 1.052)	0.249	0.024	52.30	<0.001	69.41
Study location										
North America ^2^	22	0.100 (0.024, 0.177)	0.039	0.010	0.435 (0.087, 0.782)	0.177	0.014	49.56	<0.001	57.62
Breed										
European beef cattle ^3^	25	0.119 (0.057, 0.181)	0.032	<0.001	0.491 (0.226, 0.755)	0.135	<0.001	54.51	<0.001	55.97
Grouping method for feeding										
Individual	20	0.100 (0.026, 0.174)	0.038	0.008	0.327 (0.074, 0.581)	0.129	0.011	46.76	<0.001	59.37
Pen	13	0.080 (−0.005, 0.166)	0.043	0.064	0.455 (−0.065, 0.976)	0.266	0.086	25.62	0.012	53.16
Corn silage in diet										
No	15	0.024 (−0.023, 0.071)	0.024	0.311	0.141 (−0.080, 0.362)	0.113	0.211	10.04	0.759	0.00
Yes	18	0.142 (0.061, 0.223)	0.041	0.001	0.607 (0.247, 0.967)	0.184	0.001	52.39	<0.001	67.55
Feeding period (days)										
≤80	22	0.101 (0.044, 0.159)	0.029	0.001	0.377 (0.125, 0.630)	0.129	0.003	41.71	0.005	49.65
>80	11	0.089 (−0.025, 0.203)	0.058	0.128	0.338 (−0.151, 0.827)	0.249	0.175	27.49	0.002	63.62
SRU dosage (% DM diet)										
≤1.00	22	0.081 (0.026, 0.137)	0.028	0.004	0.399 (0.167, 0.631)	0.118	0.001	34.32	0.034	38.80
>1.00	11	0.109 (−0.038, 0.256)	0.075	0.145	0.403 (−0.107, 0.913)	0.260	0.122	32.20	<0.001	68.94
Sex										
Steers	28	0.091 (0.029, 0.153)	0.032	0.004	0.372 (0.098, 0.646)	0.140	0.008	64.172	<0.001	57.93

^1^—Studies were stratified into group and subgroups by study factors that could influence performance outcome. Subgroups with <10 comparisons are excluded from analysis. ^2^—Studies were conducted in USA and Mexico. ^3^—These include: Charolais, Limousine, Hereford, Angus and Angus crossbred. SRU: slow-release urea; RMD: raw mean difference and its associated 95% confidence interval; SMD: standardised mean difference and its associated 95% confidence interval; SE: standard error. *Q*: chi-squared statistic and associated significance level (*p*-value); *I^2^*: percentage of variation.

**Table 4:** Summary of effect size estimates for feed efficiency (kg live weight gain (LWG)/kg dry matter intake (DMI)) of beef cattle fed control and SRU diets in a random-effect meta-analysis.

Group/Sub-Group ^1^	Number of Comparisons	Effect Size Estimates						Heterogeneity Tests		
		RMD (95% CI)	SE	*p*-Value	SMD (95% CI)	SE	*p*-Value	*Q*	*p*-Value	*I^2^* (%)
All trials	32	0.012 (0.005, 0.019)	0.003	<0.001	0.908 (0.417, 1.400)	0.251	*<0.001*	289.85	<0.001	89.31
Production phase										
Growing	15	0.018 (0.001, 0.035)	0.009	0.036	1.303 (0.366, 2.240)	0.478	0.006	150.81	<0.001	90.72
Finishing	17	0.008 (0.002, 0.014)	0.003	0.012	0.775 (0.279, 1.271)	0.253	0.002	98.08	<0.001	83.69
Peer-review										
No	15	0.007 (0.000, 0.014)	0.004	0.037	0.586 (−0.037, 1.208)	0.318	0.065	152.76	<0.001	90.84
Yes	17	0.016 (0.004, 0.029)	0.006	0.008	1.372 (0.559, 2.185)	0.415	0.001	116.76	<0.001	86.30
Study location										
North America ^2^	22	0.014 (0.002, 0.026)	0.006	0.025	0.978 (0.398, 1.557)	0.296	0.001	118.72	<0.001	82.31
Breed										
European beef cattle ^3^	25	0.015 (0.006, 0.023)	0.004	0.001	1.146 (0.598, 1.695)	0.280	<0.001	207.53	<0.001	88.44
Grouping method for feeding										
Individual	20	0.012 (0.003, 0.021)	0.004	0.007	0.900 (0.258, 1.542)	0.327	0.006	255.00	<0.001	92.55
Pen	12	0.012 (0.004, 0.021)	0.004	0.005	0.909 (0.238, 1.581)	0.342	0.008	32.43	0.001	66.09
Corn silage in diet										
No	14	0.006 (−0.000, 0.012)	0.003	0.054	0.469 (−0.147, 1.086)	0.315	0.136	80.17	<0.001	83.79
Yes	18	0.017 (0.006, 0.027)	0.005	0.002	1.330 (0.614, 2.045)	0.365	<0.001	180.63	<0.001	90.59
Feeding period (days)										
≤80	21	0.011 (0.005, 0.017)	0.003	<0.001	0.838 (0.298, 1.378)	0.276	0.002	169.44	<0.001	88.20
>80	11	0.014 (−0.003, 0.031)	0.009	0.114	1.269 (0.150, 2.387)	0.571	0.026	108.74	<0.001	90.803
SRU dosage (% DM diet)										
≤1.00	22	0.011	0.003	<0.001	1.032 (0.531, 1.533)	0.255	<0.001	141.91	<0.001	85.21
>1.00	10	0.014 (−0.006, 0.034)	0.010	0.164	0.615 (−0.352, 1.582)	0.493	0.212	82.89	<0.001	89.14
Sex										
Steers	27	0.013 (0.005, 0.021)	0.004	0.002	0.968 (0.397, 1.540)	0.292	0.001	233.31	<0.001	88.85

^1^—Studies were stratified into group and subgroups by study factors that could influence performance outcome. Subgroups with <10 comparisons are excluded from analysis. ^2^—Studies were conducted in USA and Mexico. ^3^—These include: Charolais, Limousine, Hereford, Angus and Angus crossbred. SRU: slow-release urea; RMD: raw mean difference and its associated 95% confidence interval; SMD: standardised mean difference and its associated 95% confidence interval; SE: standard error. *Q*: chi-squared statistic and associated significance level (*p*-value); *I^2^*: percentage of variation.

**Table 5:** Economic and environmental impacts of feeding slow-release urea (SRU) in growing-finishing beef cattle production.

Item	Baseline	SRU	Difference	% Change
Economic impact analysis ^1^				
Ration required to gain 200 kg LW (kg DM/head)	1282.05	1204.82	−77.23	6.0
Days on feed to slaughter (d)	143	134	−9	6.3
Total feed use (kg as-fed/head)	1831.50	1721.17	−110.33	6.0
Feed cost (€/head)	274.73	258.18	−16.55	6.0
Total feed cost (€/1000 head)	274,730	258,180	−16,550	6.0
Environmental impact analysis				
Emission intensity attributed to feed use (kg CO_2_-eq per beef protein output per head) ^2^	1857.6	1746.1	111.5	6.0
Emission intensity (kg CO_2_-eq per beef protein output per head)	5160	5048.5	111.5	2.2
Total emission intensity (tonnes CO_2_-eq per beef protein output per 1000 head)	5160	5048.5	111.5	2.2

^1^—This analysis considers only the economic benefit derived from a reduction in feed cost due to the positive effect of SRU on beef cattle performance. ^2^—Emission intensity is calculated relative to the beef protein output. Data on the global average emission intensity of beef was reported as 300 kg CO_2_-eq per kg of protein and an average of 36% of beef emissions was attributed to feed use [20]. This data was used to calculate the global average emission intensity of beef attributed to feed use as 108 kg CO_2_-eq per kg of protein. Thus, emission intensity attributed to feed use = Beef protein output × 108. For SRU diet, the emission intensity attributed to feed use was corrected by the −6% reduction in simulated feed use.

## Supplementary Materials

The following are available online at https://www.mdpi.com/2076-2615/10/4/657/s1. Table S1. Reference list of studies excluded from the meta-analysis. Figure S1: Forest plot of the effect of slow-release urea supplementation on dry matter intake (DMI, kg/d) of growing and finishing beef cattle. Figure S2: Forest plot of the effect of slow-release urea supplementation on dietary protein intake (DPI, kg/d) of growing and finishing beef cattle. Figure S3: Forest plot of the effect of slow-release urea supplementation on live weight gain (LWG, kg/d) of growing and finishing beef cattle. Figure S4: Forest plot of the effect of slow-release urea supplementation on feed efficiency (FE, kg LWG/kg DMI) of growing and finishing beef cattle.

## Appendix A

**Table A1:** Description of studies used in the meta-analysis examining the effect of control (CON) and slow-release urea (SRU)-supplemented diets on the performance outcome of beef cattle.

Reference	Location	Source	Breed	Sex	Feeding Regiment	Production Phase	Grouping Method	SRU Dosage (% DM Diet)	Feeding Period
Agovino et al. [45]	Ireland	Conference poster	Unknown	Heifers	Corn silage-based diet	Finishing	Animal	0.73	80
Cabrita [46]	Portugal	Conference poster	Charolais × Limousine	Heifers	Corn-based diet	Finishing	Animal	0.45	60
Corte et al. [24]	Brazil	Journal	Nellore	Steers	Sugarcane silage and baggase/corn-based diet	Finishing	Animal	1.80	75
Eweedah et al. [25]	Egypt	Journal	Holstein	Steers	Corn silage-based diet	Growing	Animal	0.61, 0.84	105
Ferres et al. [47]	Uruguay	Conference poster	Hereford	Steers	Corn silage-based diet	Finishing	Animal	0.52	65
Kononoff et al. [48]	USA	Journal	Holstein	Heifers	Corn silage/Timothy hay-based diet	Growing	Animal	1.28, 1.78	140
Muro et al. [49]	Argentina	Conference poster	Holstein	Heifers	Corn-based diet/grass hay	Growing	Animal	1.23	60
Pinos-Rodríguez et al. [50]	Mexico	Journal	Brown Swiss × Brahman	Steers	Sorghum-based diet	Finishing	Animal	1.10	48
Sgoifo Rossi et al. [51]	Italy	Conference poster	Charolais	Steers	Corn silage-based diet	Finishing	Animal	0.42	100
Simeone et al. [52]	Uruguay	Conference poster	Hereford	Steers	Sorghum-based diet/ryegrass hay	Growing and finishing	Animal	1.0, 1.5	50
Tedeschi et al. [27]	USA	Journal	Angus crossbred	Steers	Corn silage-based diet	Growing and finishing	Animal	0.4, 1.2, 0.3, 0.8	84
Wahrmund and Hersom [53]	USA	Report	Angus	Steers	Bahiagrass hay	Growing	Animal	0.27, 0.25	42
Holder [54]	USA	PhD thesis	Angus crossbred	Steers	Corn silage-based diet	Finishing	Pen	0.45, 0.9, 1.35	42
Holland and Jennings [55]	USA	Conference poster	British crossbred	Steers	Corn-based diet	Finishing	Pen	0.43, 0.83	117
López-soto et al. [36]	Mexico	Journal	Zebu, Angus, Hereford, Charolais	Steers	Sorghum-based/sudangrass hay/DDGS diet	Finishing	Pen	0.80	70
Taylor-Edwards et al. [26]	USA	Journal	Angus crossbred	Steers	Corn silage-based diet	Growing	Pen	0.4, 0.8, 1.2, 1.6	56
Manella et al. [56]	Brazil	Conference poster	Nellore	Steers	Sugarcane silage-based diet	Finishing	Pen	1.80	80

**Table A2:** Simulation inputs used for the economic and environmental impacts of feeding slow-release urea (SRU) in growing-finishing beef cattle production.

Item	Baseline	SRU	Difference	% Change
Number of cattle	1000	1000		
Dry matter intake (kg DM/d/head)	9	9		
Live weight gain (LWG, kg/d/head) ^1^	1.400	1.492	+0.092	6.6
Feed efficiency (kg LWG/kg DMI/head)	0.156	0.166	+0.010	6.4
Target live weight gain (kg/head)	200	200		
Lean meat yield (kg/head) ^2^	82	82		
Beef protein output (kg/head) ^3^	17.2	17.2		

^1^—The live weight gain of beef cattle fed SRU diets was corrected based on the current meta-analysis results which showed an average increase of +92 g/d/head in the live weight gain of beef cattle fed SRU diets. ^2^—Meat yield was calculated as a proportion of the target live weight gain (200 kg). Average lean meat yield of beef cattle was assumed to be 41% of the live weight according to Holland et al. [57]. ^3^—Beef protein output was calculated as a proportion of the lean meat yield. Beef contains an average of 21% protein [58,59].
