# Supplementary material for: A Meta-Analysis of the Effects of Slow-Release Urea Supplementation on the Performance of Beef Cattle
Source: Animals (Basel). 2020 Apr 10;10(4):657. doi: 10.3390/ani10040657 (PMC7223368; doi:10.3390/ani10040657)
Supplement: Supplementary file 1 [file animals-10-00657-s001.pdf]

animals

Supplementary Files: A Meta-Analysis of the Effects of Slow-Release Urea Supplementation on the Performance of Beef Cattle

Saheed A. Salami [1,*], Colm A. Moran [2], Helen E. Warren [1] and Jules Taylor-Pickard [3]

Table S1. Reference list of studies excluded from the meta-analysis.

Almora, E. A., Huntington, G. B., & Burns, J. C. (2012). Effects of supplemental urea sources and feeding frequency on ruminal fermentation, fiber digestion, and nitrogen balance in beef steers. Animal feed science and technology, 171(2-4), 136-145.

Benedeti, P. D. B., Paulino, P. V. R., Marcondes, M. I., Valadares Filho, S. D. C., Martins, T. S., Lisboa, E. F., ... & Duarte, M. S. (2014). Soybean meal replaced by slow release urea in finishing diets for beef cattle. Livestock Science, 165, 51-60.

Bourg, B. M., Tedeschi, L. O., Wickersham, T. A., & Tricarico, J. M. (2012). Effects of a slow-release urea product on performance, carcass characteristics, and nitrogen balance of steers fed steam-flaked corn. *Journal of Animal Science*, *90*(11), 3914-3923.

Ceconi, I., Ruiz-Moreno, M. J., DiLorenzo, N., DiCostanzo, A., & Crawford, G. I. (2015). Effect of slow-release urea inclusion in diets containing modified corn distillers grains on total tract digestibility and ruminal fermentation in feedlot cattle. Journal of animal science, 93(8), 4058-4069.

Fialho, M. P. F., Miranda, P. A. B., Saliba, E. O. S., De Oliveira, L. O. F., Lopes, V., Da Silva, J. J., Moraes, S. A. D. (2007). Ruminal fermentation characteristics and microbial protein synthesis of cattle fed supplements with different combinations of carbohydrate and protein. Scientific poster presented at the 23rd Alltech Symposium, 20-23 May 2007, Lexington, USA.

Garcia-Gonzalez, R., Tricarico, J. M., Harrison, G. A., Meyer, M. D., McLeod, K. R., Harmon, D. L., & Dawson, K. A. (2007). Optigen (R) is a sustained release source of non-protein nitrogen in the rumen. J. Anim. Sci., 85(Suppl. 1), 98.

Gonçalves, A. P., Nascimento, C. F. M. D., Ferreira, F. A., Gomes, R. D. C., Manella, M. D. Q., Marino, C. T., ... & Rodrigues, P. H. M. (2015). Slow-release urea in supplement fed to beef steers. Brazilian Archives of Biology and technology, 58(1), 22-30.

Holder, V., Harmon, D., Jennings, J. (2013). The effects of basal diet and the supply of degradable intake protein on growth of steers. Scientific poster presented at the 29th Alltech Symposium, 19-22 May 2013, Lexington, USA.

Holder, V., Harmon, D., Jennings, J. (2013). Effects of replacing soybean meal N with NPN from urea or Optigen on intake and performance of receiving cattle. Scientific poster presented at the 29th Alltech Symposium, 19-22 May 2013, Lexington, USA.

Holder, V. B., El-Kadi, S. W., Tricarico, J. M., Vanzant, E. S., McLeod, K. R., & Harmon, D. L. (2013). The effects of crude protein concentration and slow release urea on nitrogen metabolism in Holstein steers. Archives of animal nutrition, 67(2), 93-103.

Holder, V. B., Tricarico, J. M., Kim, D. H., Kristensen, N. B., & Harmon, D. L. (2015). The effects of degradable nitrogen level and slow release urea on nitrogen balance and urea kinetics in Holstein steers. Animal Feed Science and Technology, 200, 57-65.

López-Soto, M. A., Aguilar-Hernández, J. A., Dávila-Ramos, H., Estrada-Angulo, A., Ríos, F. G., Urías-Estrada, J. D., ... & Plascencia, A. (2015). Effects of a combining feed grade urea and a slow-release product on performance, dietary energetics and carcass characteristics of steers fed finishing diets. Journal of Applied Animal Research, 43(3), 303-308.

Manella, M. Q., Matsuda, E. Y., & Carvalho, F.A.N. (2007). Replacing vegetable protein in supplements with Optigen II in beef steers fed sugar cane silage as the sole forage. Scientific poster presented at the 23rd Alltech Symposium, 20-23 May 2007, Lexington, USA.

Marchesin, W. A., Herling, V. R., de Moraes, A. L., Luz, P. H. C., Sanchez, J. M. D., Pereira, T. Q. M.,Manella, M. Q., Putrino, S. M., & Glossklaus, C. (2008). Rearing Nelore heifers using low and high intake supplements in winter. Scientific poster presented at the 24th Alltech Symposium, 20-23 April 2008, Lexington, USA.

Pazdiora, R. D., Resende, F. D. D., Faria, M. H. D., Siqueira, G. R., Almeida, G. B. D. S., Sampaio, R. L., ... & Prietto, M. S. R. (2013). Animal performance and carcass characteristics of Nellore young bulls fed coated or uncoated urea slaughtered at different weights. Revista Brasileira de Zootecnia, 42(4), 273-283.

Wahrmund, J., De Araujo, D. V., Hersom, M., & Arthington, J. (2007). Evaluation of Optigen® II as a source of rumen degradable protein for mature beef cows. 2007 Florida Beef Report, University of Florida, Gainesville, Florida, USA, 61-63.

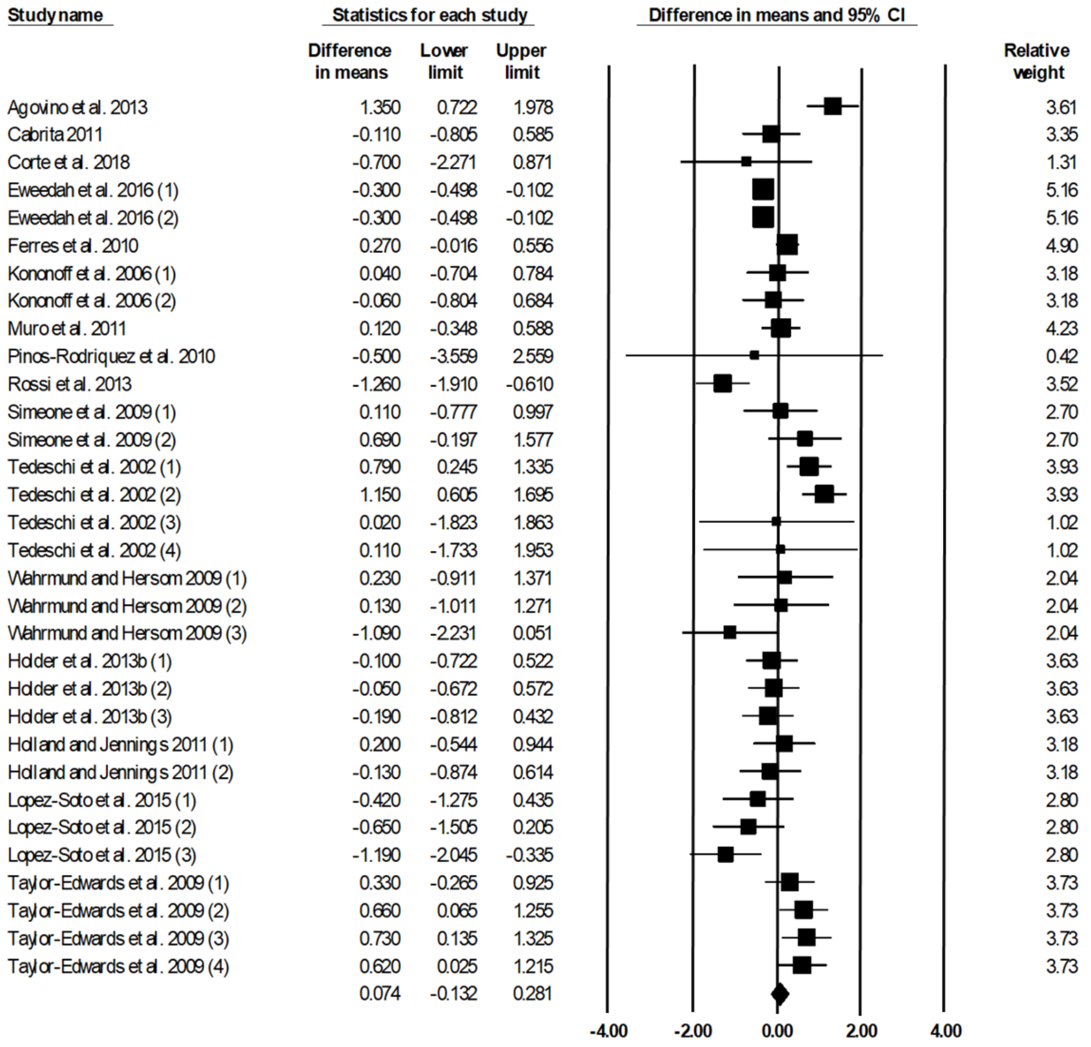

Figure S1. Forest plot of the effect of slow-release urea supplementation on dry matter intake (DMI, kg/d) of growing and finishing beef cattle. Study name refers to the reference of that study. Raw mean differences (difference in means) represent the effect size estimate. Each square represents the mean effect size for that study. Thus, the squares to the right of the zero mid-line represent an increase in DMI, squares to the left of the 0 mid-line indicate a decrease in DMI and squares at the 0 mid-line indicate no effect on DMI. The upper and lower limit of the line connected to the square represents the upper and lower 95% confidence interval (CI) for the effect size. The size of the square reflects the relative weighting of the study to the overall effect size estimate, with larger squares representing greater weight. The diamond at the bottom represents the overall effect size estimate.

Study name	Statistics for each study			Relative weight
	Difference in means	Lower limit	Upper limit	
Agovino et al. 2013	0,130	0,049	0,211	3,32
Cabrita 2011	0,020	-0,098	0,138	3,05
Corte et al. 2018	-0,140	-0,320	0,040	2,53
Eweedah et al. 2016 (1)	-0,080	-0,117	-0,043	3,54
Eweedah et al. 2016 (2)	-0,110	-0,147	-0,073	3,54
Ferres et al. 2010	-0,050	-0,087	-0,013	3,54
Kononoff et al. 2006 (1)	-0,010	-0,128	0,108	3,05
Kononoff et al. 2006 (2)	0,000	-0,118	0,118	3,05
Muro et al. 2011	0,080	0,013	0,147	3,40
Pinos-Rodriquez et al. 2010	0,000	-0,386	0,386	1,22
Rossi et al. 2013	-0,400	-0,494	-0,306	3,23
Simeone et al. 2009 (1)	0,010	-0,090	0,110	3,18
Simeone et al. 2009 (2)	0,060	-0,026	0,146	3,28
Tedeschi et al. 2002 (1)	0,130	0,086	0,174	3,51
Tedeschi et al. 2002 (2)	0,280	0,225	0,335	3,47
Tedeschi et al. 2002 (3)	0,060	-0,104	0,224	2,67
Tedeschi et al. 2002 (4)	0,180	0,006	0,354	2,58
Wahrmund and Hersom 2009 (1)	0,140	-0,008	0,288	2,80
Wahrmund and Hersom 2009 (2)	0,130	-0,003	0,263	2,92
Wahrmund and Hersom 2009 (3)	-0,170	-0,311	-0,029	2,86
Holder et al. 2013b (1)	-0,050	-0,129	0,029	3,33
Holder et al. 2013b (2)	-0,080	-0,159	-0,001	3,33
Holder et al. 2013b (3)	-0,140	-0,219	-0,061	3,33
Holland and Jennings 2011 (1)	0,030	-0,057	0,117	3,28
Holland and Jennings 2011 (2)	-0,010	-0,097	0,077	3,28
Lopez-Soto et al. 2015 (1)	0,130	0,006	0,254	3,00
Lopez-Soto et al. 2015 (2)	0,090	-0,034	0,214	3,00
Lopez-Soto et al. 2015 (3)	0,000	-0,124	0,124	3,00
Taylor-Edwards et al. 2009 (1)	0,080	0,018	0,142	3,43
Taylor-Edwards et al. 2009 (2)	0,170	0,108	0,232	3,43
Taylor-Edwards et al. 2009 (3)	0,240	0,178	0,302	3,43
Taylor-Edwards et al. 2009 (4)	0,280	0,218	0,342	3,43
	0,033	-0,020	0,085	

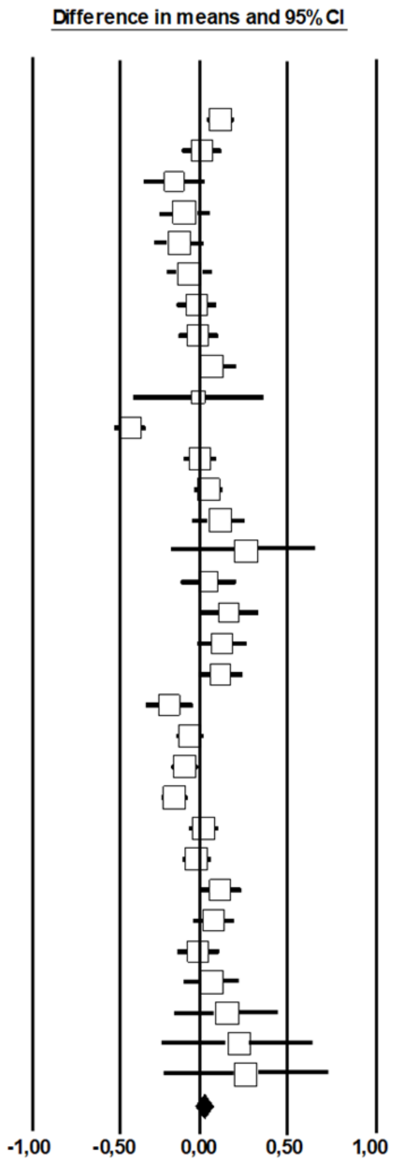

Figure S2. Forest plot of the effect of slow-release urea supplementation on dietary protein intake (DPI, kg/d) of growing and finishing beef cattle. Study name refers to the reference of that study. Raw mean differences (difference in means) represent the effect size estimate. Each square represents the mean effect size for that study. Thus, the squares to the right of the zero mid-line represent an increase in DPI, squares to the left of the 0 mid-line indicate a decrease in DPI and squares at the 0 mid-line indicate no effect on DPI. The upper and lower limit of the line connected to the square represents the upper and lower 95% confidence interval (CI) for the effect size. The size of the square reflects the relative weighting of the study to the overall effect size estimate, with larger squares representing greater weight. The diamond at the bottom represents the overall effect size estimate.

Study name	Statistics for each study			Relative weight
	Difference in means	Lower limit	Upper limit	
Agovino et al. 2013	0.230	0.088	0.372	3.86
Cabrita 2011	0.120	-0.037	0.277	3.15
Corte et al. 2018	-0.100	-0.411	0.211	0.80
Eweedah et al. 2016 (1)	0.040	-0.072	0.152	6.23
Eweedah et al. 2016 (2)	-0.100	-0.212	0.012	6.23
Ferres et al. 2010	0.210	0.145	0.275	18.62
Kononoff et al. 2006 (1)	0.000	-0.198	0.198	1.97
Kononoff et al. 2006 (2)	-0.070	-0.268	0.128	1.97
Muro et al. 2011	0.200	-0.118	0.518	0.77
Pinos-Rodriquez et al. 2010	-0.100	-0.687	0.487	0.22
Rossi et al. 2013	0.090	-0.057	0.237	3.60
Simeone et al. 2009 (1)	-0.080	-0.280	0.120	1.93
Simeone et al. 2009 (2)	0.080	-0.120	0.280	1.93
Tedeschi et al. 2002 (1)	0.280	0.106	0.454	2.55
Tedeschi et al. 2002 (2)	0.590	0.416	0.764	2.55
Tedeschi et al. 2002 (3)	0.110	-0.239	0.459	0.64
Tedeschi et al. 2002 (4)	0.120	-0.229	0.469	0.64
Wahrmund and Hersom 2009 (1)	0.020	-0.143	0.183	2.92
Wahrmund and Hersom 2009 (2)	0.050	-0.113	0.213	2.92
Wahrmund and Hersom 2009 (3)	0.100	-0.063	0.263	2.92
Holder et al. 2013b (1)	-0.050	-0.288	0.188	1.37
Holder et al. 2013b (2)	0.020	-0.218	0.258	1.37
Holder et al. 2013b (3)	-0.040	-0.278	0.198	1.37
Holland and Jennings 2011 (1)	0.020	-0.104	0.144	5.05
Holland and Jennings 2011 (2)	-0.040	-0.164	0.084	5.05
Lopez-Soto et al. 2015 (1)	0.120	-0.078	0.318	1.97
Lopez-Soto et al. 2015 (2)	0.000	-0.198	0.198	1.97
Lopez-Soto et al. 2015 (3)	-0.160	-0.358	0.038	1.97
Taylor-Edwards et al. 2009 (1)	0.150	-0.011	0.311	2.99
Taylor-Edwards et al. 2009 (2)	0.330	0.169	0.491	2.99
Taylor-Edwards et al. 2009 (3)	0.350	0.189	0.511	2.99
Taylor-Edwards et al. 2009 (4)	0.180	0.019	0.341	2.99
Manella et al. 2007	0.070	-0.156	0.296	1.51
	0.107	0.080	0.135	

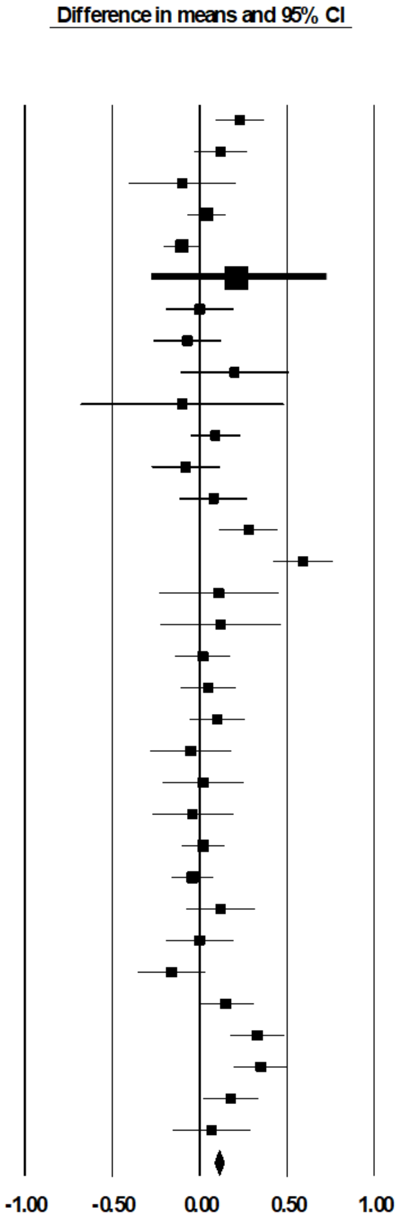

Figure S3. Forest plot of the effect of slow-release urea supplementation on liveweight gain (LWG, kg/d) of growing and finishing beef cattle. Study name refers to the reference of that study. Raw mean differences (difference in means) represent the effect size estimate. Each square represents the mean effect size for that study. Thus, the squares to the right of the zero mid-line represent an increase in LWG, squares to the left of the 0 mid-line indicate a decrease in LWG and squares at the 0 mid-line indicate no effect on LWG. The upper and lower limit of the line connected to the square represents the upper and lower 95% confidence interval (CI) for the effect size. The size of the square reflects the relative weighting of the study to the overall effect size estimate, with larger squares representing greater weight. The diamond at the bottom represents the overall effect size estimate.

Study name	Statistics for each study			Relative weight
	Difference in means	Lower limit	Upper limit	
Agovino et al. 2013	0.010	0.005	0.015	3.36
Cabrita 2011	0.020	0.014	0.026	3.35
Corte et al. 2018	0.000	-0.001	0.001	3.42
Eweedah et al. 2016 (1)	0.010	0.004	0.016	3.33
Eweedah et al. 2016 (2)	-0.010	-0.016	-0.004	3.33
Ferres et al. 2010	0.020	0.018	0.022	3.41
Kononoff et al. 2006 (1)	0.000	-0.006	0.006	3.33
Kononoff et al. 2006 (2)	-0.010	-0.016	-0.004	3.33
Muro et al. 2011	0.030	0.016	0.044	2.98
Pinos-Rodriquez et al. 2010	0.000	-0.017	0.017	2.84
Rossi et al. 2013	0.020	0.015	0.025	3.35
Simeone et al. 2009 (1)	-0.020	-0.027	-0.013	3.30
Simeone et al. 2009 (2)	0.000	-0.007	0.007	3.30
Tedeschi et al. 2002 (1)	0.040	0.036	0.044	3.38
Tedeschi et al. 2002 (2)	0.080	0.075	0.085	3.35
Tedeschi et al. 2002 (3)	0.010	0.002	0.018	3.28
Tedeschi et al. 2002 (4)	0.010	0.002	0.018	3.28
Wahrmund and Hersom 2009 (1)	0.000	-0.012	0.012	3.10
Wahrmund and Hersom 2009 (2)	0.000	-0.012	0.012	3.10
Wahrmund and Hersom 2009 (3)	0.030	0.018	0.042	3.10
Holder et al. 2013b (1)	-0.010	-0.035	0.015	2.35
Holder et al. 2013b (2)	0.000	-0.025	0.025	2.35
Holder et al. 2013b (3)	0.000	-0.025	0.025	2.35
Holland and Jennings 2011 (1)	0.000	-0.007	0.007	3.29
Holland and Jennings 2011 (2)	0.000	-0.007	0.007	3.29
Lopez-Soto et al. 2015 (1)	0.020	0.009	0.031	3.13
Lopez-Soto et al. 2015 (2)	0.010	-0.001	0.021	3.13
Lopez-Soto et al. 2015 (3)	0.000	-0.011	0.011	3.13
Taylor-Edwards et al. 2009 (1)	0.020	0.005	0.035	2.94
Taylor-Edwards et al. 2009 (2)	0.040	0.025	0.055	2.94
Taylor-Edwards et al. 2009 (3)	0.040	0.025	0.055	2.94
Taylor-Edwards et al. 2009 (4)	0.020	0.005	0.035	2.94
	0.012	0.005	0.019	

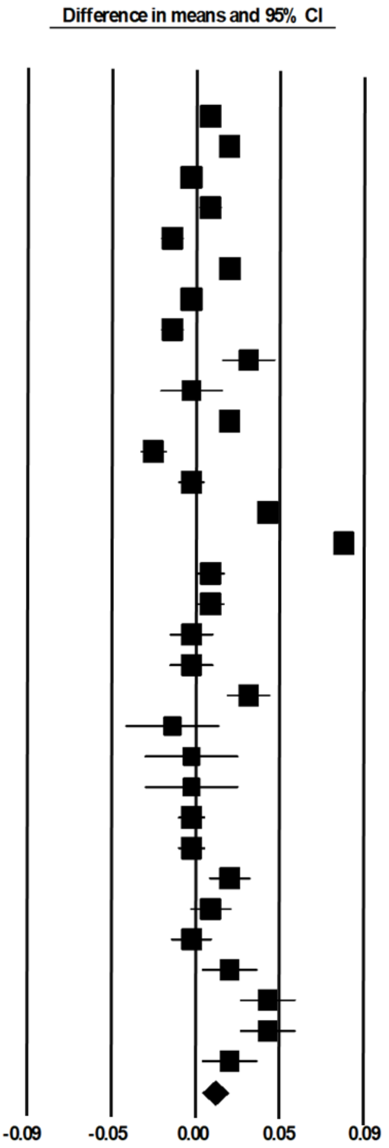

Figure S4. Forest plot of the effect of slow-release urea supplementation on feed efficiency (FE, kg LWG/kg DMI) of growing and finishing beef cattle. Study name refers to the reference of that study. Raw mean differences (difference in means) represent the effect size estimate. Each square represents the mean effect size for that study. Thus, the squares to the right of the zero mid-line represent an increase in FE, squares to the left of the 0 mid-line indicate a decrease in FE and squares at the 0 mid-line indicate no effect on FE. The upper and lower limit of the line connected to the square represents the upper and lower 95% confidence interval (CI) for the effect size. The size of the square reflects the relative weighting of the study to the overall effect size estimate, with larger squares representing greater weight. The diamond at the bottom represents the overall effect size estimate.

© 2020 by the authors. Licensee MDPI, Basel, Switzerland. This article is an open access article distributed under the terms and conditions of the Creative Commons Attribution (CC BY) license (http://creativecommons.org/licenses/by/4.0/).
